# From the mechanism of action to clinical management: A review of cardiovascular toxicity in adult treated with CAR-T therapy

**DOI:** 10.1016/j.htct.2024.06.008

**Published:** 2024-09-07

**Authors:** Frank Nunes, Breno Moreno de Gusmão, Franciely Bueno Wiginesk, Euler Manenti, Juliana Soares, Mizianne Garcia Freitas, Juliane Dantas Seabra-Garcez, Alexandre Manoel Varela, João Pedro Passos Dutra, Bruno Cesar Bacchiega, Tânia Félix Lorenzato da Fonseca Peixoto, Carolina Maria Pinto Domingues de Carvalho e Silva, Renato D. Lopes, Ariane Vieira Scarlatelli Macedo

**Affiliations:** a Hospital César Leite, Manhuaçu, Brazil; b Hospital BP, São Paulo, Brazil; c Instituto de Cardiologia Campo Grande, Campo Grande, Brazil; d Instituto de Medicina Vascular, Hospital Mãe de Deus, Porto Alegre, Brazil; e Hospital Israelita Albert Einstein, São Paulo, Brazil; f Prefeitura de Campo Grande, Campo Grande, Brazil; g Hospital São Lucas Rede D'Or São Luiz, Aracaju, Brazil; h Hospital Primavera, Aracaju, Brazil; i Hospital Universitário Evangélico Mackenzie, Curitiba, Brazil; j Complexo Hospital de Clínicas da Universidade Federal do Paraná, Curitiba, Brazil; k Hospital Erasto Gaertner, Curitiba, Brazil; l Centro de Pesquisas Oncológicas, Florianópolis, Brazil; m Hospital SOS Cárdio, Florianópolis, Brazil; n Barretos Cancer Hospital, Barretos, Brazil; o Hospital Felício Rocho, Belo Horizonte, Brazil; p CardioPrime Cardiovascular Center, Ribeirão Preto, Brazil; q Duke University /Duke Clinical Research Institute, Durham, USA; r Faculdade de Ciências Médicas da Santa Casa de São Paulo, São Paulo, Brazil

**Keywords:** Cardiotoxicity, Chimeric antigen receptor therapy, Cytokine release syndrome, Cardio-Oncology, Hematology

## Abstract

Chimeric antigen receptor T-cell therapy represents an innovative approach to immunotherapy and currently stands out, particularly for oncohematological patients refractory to traditional treatments. Ongoing trials are further expanding its clinical use for new oncological and non-oncological indications, potentially leading to newer treatment options soon.

This new approach, however, also presents challenges, including cardiovascular toxicity. Little is reported in pivotal studies, and some recent retrospective observations suggest a non-negligible incidence of side effects with presentation ranging from mild adverse cardiovascular events to fatal complications in which, in most cases, there is a direct or indirect association with cytokine release syndrome.

In this literature review, the hypotheses of an important interface between cytokine release syndrome and cardiotoxicity by chimeric antigen receptor T-cell therapy will be addressed, as will current knowledge about risk factors for cardiotoxicity and recommendations for pre-therapy evaluation, post-infusion monitoring and clinical management of these complications.

## Introduction

Several mechanisms, essential for the emergence and development of different types of cancer, interact in the pathogenesis of cancer, altering the functional capabilities of tumor cells in relation to normal cells. Resistance to cell death, reprogramming of cellular metabolism, sustained proliferative signaling, inflammation promoted by the tumor, genomic instability and mutation, capacity for neoangiogenesis, and ability to escape destruction by the immune system are examples of these newly acquired functions.^1^

Acting at strategic points in these mechanisms of cancer pathogenesis, new forms of treatment have emerged in recent decades, such as in relation to intracellular signaling pathways and to counter tumor escape from the immune system. Targeted therapies and immunotherapy have revolutionized the treatment landscape for both solid and hematological malignancies, complementing traditional approaches like surgery, radiotherapy, and chemotherapy.

The general principle of immunotherapy consists of reactivating a preexisting immune response that was paralyzed by the tumor's ability to escape the host's immune response or a therapy with the capacity to act through a *de novo* immune response against the neoplasia. Immune checkpoint inhibitors are the best known of the immunological therapies, while vaccines against tumor neoantigens and chimeric antigen receptor (CAR)-T cell therapy are examples of other routes of action to combat tumors through the immune system.^2^

Although CAR-T cell therapy is disruptive in nature, significantly improving the prognosis of adult and pediatric oncohematological patients, many of whom have advanced and refractory disease, it is not free from adverse effects. Cytokine release syndrome (CRS), immune effector cell-associated neurotoxicity syndrome (ICANS), infections, myelosuppression and bleeding disorders are examples of adverse effects, in some cases with fatal outcomes. In November 2023, the US Food and Drug Administration (FDA) announced rare cases of secondary malignancies after CAR-T cell therapy, including CAR-positive lymphoma.^3^^,^^4^ Despite great uncertainty to date, in light of current knowledge, this risk is extremely low, and the benefits of CAR-T therapy may outweigh this risk.

Cardiovascular toxicity has also been reported since the first pivotal studies of CAR-T cell treatment in oncohematological patients. In the pediatric population, cardiotoxicity appears to be self-limited in retrospective cohorts, with recovery of systolic function within 3–6 months.^5^^,^^6^ However, in adults, the clinical picture can vary from subclinical or mild manifestations to fatal complications.

This review aims to summarize current knowledge on cardiovascular toxicity related to CAR-T cell therapy in adults, encompassing its pathophysiological mechanism, clinical characteristics, risk stratification measures in pre-therapy assessments, monitoring, and treatment of this complication in order to reduce the impact it causes on the morbidity and mortality of adult patients undergoing this type of immunotherapy.

### Chimeric antigen receptor T-cell therapy

CAR-T cell therapy is an innovative type of immunotherapy that stimulates a direct antitumor response via a cell therapy mechanism through *ex vivo* genetically modified T-cells.^2^

T-cells play an essential role in the immune system, defending against disease-causing pathogens. They also protect the body against abnormal cells, such as tumor cells. However, one of the mechanisms supporting tumor development is the ability of cells to avoid tumor destruction via the immune system.^1^

In CAR-T cell therapy, autologous T-cells isolated from peripheral blood are genetically modified *ex vivo* via viral vectors (lentivirus or retrovirus) to express an artificial chimeric antigen receptor. The chimeric antigen receptor is a synthetic transmembrane protein expressed on the surface of immune effector cells, which are reprogrammed to allow simultaneous detection of surface antigens and activation of T-cells without the need for antigen presentation by the major histocompatibility complex. Before being reinfused, the patient receives conditioning chemotherapy, generally with fludarabine phosphate (Fludarabine Phosphate) and cyclophosphamide.^2^^,^^7^

The main purposes of lymphodepleting chemotherapy are (1) the reduction of endogenous lymphocytes to prepare a niche for the engraftment of CAR-T infusions and to support their long-term activity; (2) reduction of tumor cells to avoid rapid exhaustion of CAR-T cells; (3) preparation and reprogramming of the microenvironment and soluble factors to ensure optimal engraftment, homing, and long-term survival of CAR-T cells.^2^

**Figure 1:**
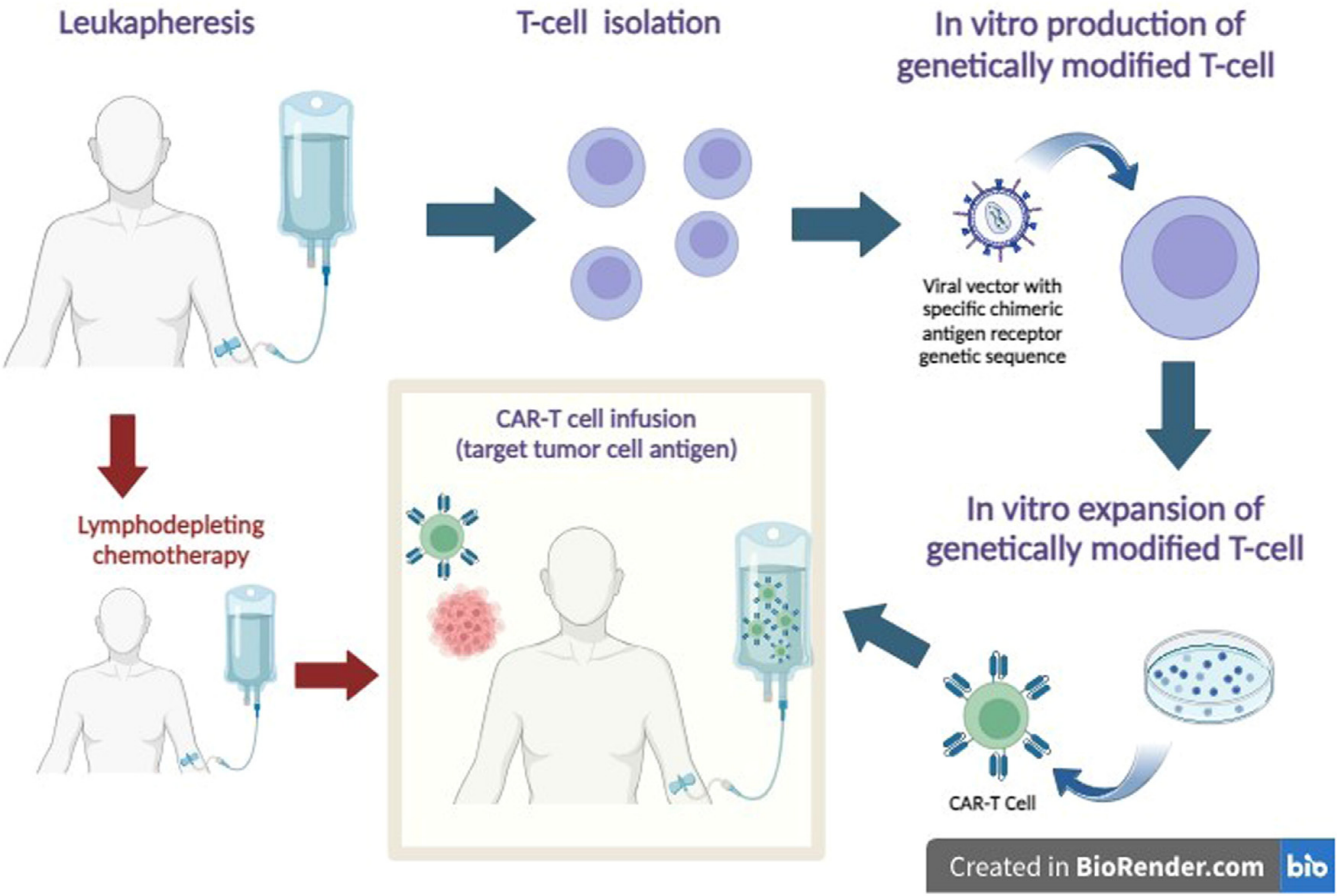
CAR-T Cell Therapy. After leukapheresis, T cells are isolated, and a tumor-specific chimeric antigen receptor is inserted into the cell surface using a viral vector. An expansion of cell mass is promoted in a culture medium. To receive genetically modified T-cells, the patient previously receives lymphodepleting chemotherapy. After infusion, CAR-T cells recognize the tumor antigen and act to promote tumor cell death.

After being reinfused, these redesigned lymphocytes, expand and act on target antigens of tumor cells, especially those that show increased levels of specific antigens, such as the CD19 antigen, which is overexpressed in B-cell neoplasms, for example acute lymphoblastic leukemia and non-Hodgkin's lymphoma (Figure 1).^2^^,^^7^^,^^8^

The first attempt to therapeutically use T-cells with genetically modified antigen receptors was to treat people infected with human immunodeficiency virus (HIV). However, the infusion of CAR-T cells was unsuccessful in reducing viral load.^9^^,^^10^ In the oncohematological area, since the publication in 2011 of the first case report of efficacy in a patient with chronic lymphoid leukemia, the list of indications of CAR-T cell therapy has expanded and proven to be revolutionary, especially in patients with advanced disease by radically modifying previously unfavorable prognoses.^11^ The main CAR-T products and their respective indications in adult and pediatric oncohematological patients are described in Table 1.^12^

**Table 1:** Chimeric antigen receptor (CAR) T-cell products and indications.

Generic Name	Indication (relapsed or refractory disease)
Tisagenlecleucel	Large B-cell lymphoma, follicular lymphoma, pediatric or young-adult B-cell acute lymphoblastic leukemia
Axicabtagene ciloleucel	Large B-cell lymphoma
Brexucabtagene autoleucel	B-cell acute lymphoblastic leukemia, mantle-cell lymphoma
Idecabtagene vicleucel	Multiple Myeloma
Lisocabtagene maraleucel	Large B-cell lymphoma, follicular lymphoma, primary mediastinal large B-cell lymphoma
Ciltacabtagene autoleucel	Multiple myeloma

Despite the little data currently available, the durability of the immune response triggered by genetically modified T-cells appears to be prolonged, as reported in a recent publication in which two patients with chronic lymphoid leukemia achieved complete remission in 2010 with CAR-T cell therapy and, after more than ten years, they remain with detectable, active CAR-T cells and maintain leukemia remission.^13^

### Cardiovascular adverse events of CAR-T cell therapy: the interplay between cytokine release syndrome and cardiotoxicity

CRS is the most common adverse effect of CAR-T cell therapy, present in the majority of patients. In a retrospective real-world pharmacovigilance study of the 2657 CAR-T recipient safety reports, CRS was reported in 1457 patients (54.8 %).^14^ In the controlled clinical trial environment, CRS is reported at even higher rates such as in the recent CARTITUDE-4 trial of patients with multiple myeloma, where CRS was reported in 76.1 % of the patients.^15^

Among the cytokines released, the elevation of interleukin (IL)−1 and IL-6, especially the latter, stands out, as their levels are directly related to the severity of symptoms.^16^

CRS develops because of hyperactivation of the immune system. After reinfusion into the patient, T-cells with chimeric antigen receptors recognize the tumor target antigen, promoting the release of pro-inflammatory cytokines into the tumor microenvironment, including IL-1, IL-2 receptors, IL-6, interferon-gamma (IFN-γ), and tumor necrosis factor-alpha (TNF-∝) as part of the immune activation mechanism originated from the infused cells themselves and from other local immune cells like monocytes and macrophages.^7^^,^^17^ In most hosts, there is a massive and acute release of these circulating cytokines that promotes a systemic inflammatory response called CRS.

The main signs and symptoms of CRS are fever, myalgia, weakness, and arthralgia; in more severe cases, it is associated with hypotension and hypoxia. The most commonly used grading system to stratify severity and guide management is that proposed by the American Society for Transplantation and Cellular Therapy (ASTCT) as detailed in Table 2.^18^

**Table 2:** Cytokine release syndrome grading system and management - the American society for transplantation and cellular therapy consensus.

Presentation	Grade 1	Grade 2	Grade 3	Grade 4
Fever (>38 °C) ± constitutional symptoms (myalgia, arthralgia, malaise)	+	+	+	+
⇑⇑⇑⇑ Hydration and symptomatic treatment				
With				
Hypotension	–	+ No vasopressors	+ Vasopressor needed with/without vasopressin	+ Multiple vasopressor needed excluding vasopressin
Or				
Hypoxia	-	+ Low-flow nasal cannula	+ High-flow nasal cannula, facemask, Venturi mask	+ Positive pressure (CPAP, BiPAP, intubation + mechanical ventilation)
⇑⇑⇑ Tocilizumab and in refractory cases ⇒ combine corticosteroids				

BiPAP: bilevel positive airway pressure; CPAP: continuous positive airway pressure; CRS: cytokine release syndrome.

CRS is one of the potential mechanisms of cardiotoxicity through the action of several cytokines. IL-6 is the primary driver of the inflammation activation cascade by stimulating increased activity of B-cells and T-cells and the release of acute phase reactive proteins. This hyperactivation triggers the coagulation cascade with consequent coagulopathy, in addition to vascular leakage, microvascular dysfunction, and cardiomyopathy. TNF-∝ and IFN-γ are associated with endothelial injury, hypotension, and heart failure.^19^^,^^20^

In a retrospective real-world pharmacovigilance study, cardiotoxicity and CRS overlapped by over 60 %.^14^ Although the pathophysiology of CRS has not yet been completely elucidated, several causal factors have been postulated. First, both share some pathophysiological mechanisms. Second, both share some risk factors. Third, CRS is considered one of the potential mechanisms for cardiotoxicity. Finally, some clinical signs, such as sinus tachycardia and arterial hypotension, are used as diagnostic criteria for both adverse effects.^7^

Other possible mechanisms involved in this complex pathogenesis are ‘on-target, off-tumor’ and ‘off-target, off-tumor’ toxicities. In ‘on-target, off-tumor’ toxicity, there is an attack by T-cells on normal tissue cells that express tumor-associated antigens.^20^ In ‘off-tumor, off-target’ toxicity, there is a cross-reactivity of CAR-T cells against the titin protein, which has an epitope very similar to MAGE-A3, an epitope widely expressed in myeloma cells.^21^

**Figure 2:**
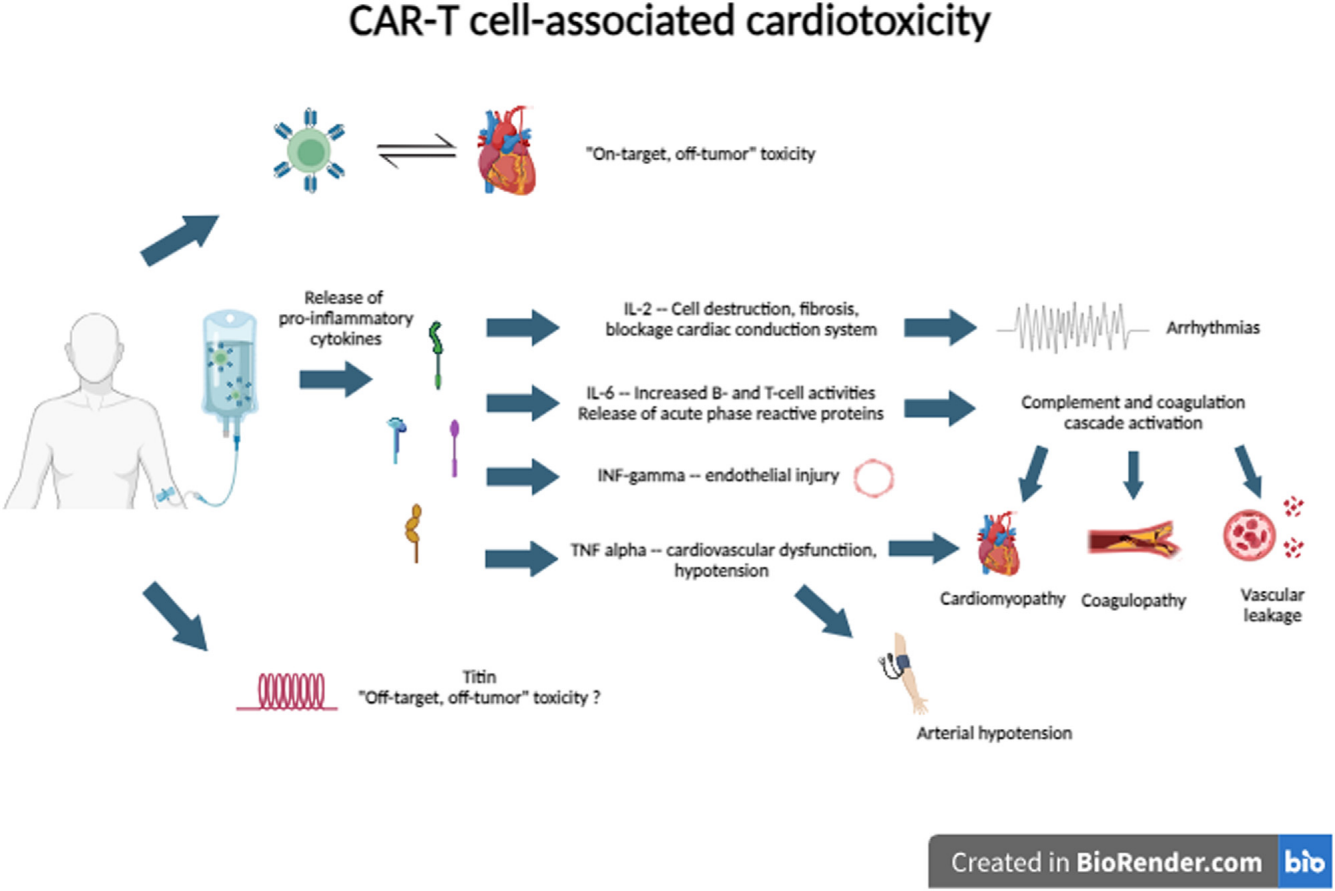
CAR-T cell associated cardiotoxicity. IL: interleukin.

The potential cardiotoxicity mechanisms of CAR-T cell therapy are described in Figure 2.

The most frequent cardiovascular adverse effects (CVAE) described are: arterial hypotension, sinus tachycardia, elevation of serum troponin levels, left ventricular dysfunction, heart failure, arrhythmias and cardiovascular death.^22^ Cardiovascular complications are predominantly reported in patients with CRS grade ≥ 2. Other conditions associated with a higher risk of CVAE are related to the recipient and the tumor as described in Table 3.^8^^,^^16^^,^^23^

**Table 3:** Conditions associated with increased risk for adverse cardiovascular outcomes.

Presence of cardiovascular risk factors, such as hyperlipidemia, hypertension, diabetes, and smoking
Previous exposure to cardiotoxic chemotherapy
Previous exposure to radiation
Baseline cardiac disease such as heart failure, moderate to severe valvular heart disease, coronary artery disease and arrhythmias
Tumor burden
High intensity lymphodepleting treatment
High CAR-T dose

Although CRS can occur at any time post-infusion, the average time between infusion and occurrence is from 2 to 5 days with the vast majority within three weeks. Likewise, cardiotoxicity predominates within the first three weeks.^7^^,^^16^^,^^24^

In pivotal studies of CAR-T cell therapy in adults, the prevalence of tachycardia and hypotension ranged from 11 to 41 % and 22–26 %, respectively.^25^, ^26^, ^27^ The characterization of hypotension and tachycardia as CVAE is controversial as they are epiphenomena of capillary leak and CRS. The overlap between CRS and CVAE was greater than 60 % in a pharmacovigilance study with over 2600 adult patients exposed to CAR-T cell therapy.^14^ However, according to this same study, 31.7 % of cardiovascular and pulmonary adverse events occurred independently of CRS.

Severe CVAE are rarely reported in pivotal studies.^25^, ^26^, ^27^, ^28^, ^29^ However, real-world data suggest that CVAE is common in patients after CAR-T cell therapy. An observational cohort evaluating 137 adult patients for the occurrence of CVAE,^24^ cardiotoxicity characterized by elevated troponin occurred in 29 of 53 tested patients (54 %), and a decreased left ventricular ejection fraction in 8 of 29 (28 %). In this study, cardiovascular events were a composite of arrhythmias, decompensated heart failure and cardiovascular death and occurred in 12 % of cases, all with grade ≥ 2 CRS. Data from a real-world pharmacovigilance study reported severe CVAE, tachyarrhythmias, cardiomyopathies and cardiogenic shock in 6.1 %, 2.79 %, 2.6 % and 1.84 % of patients, respectively.^14^ A meta-analysis of 25 studies demonstrated pooled incidence rates of overall cardiovascular events, arrhythmias, and cardiovascular dysfunction of 25.6 %, 19.2 %, and 8 %, respectively.^20^ The difference between the incidence rates of these real-world studies and pivotal trials is probably related to the fact that the latter have been underpowered to detect adverse cardiovascular events for some reasons such as small samples, patients with known cardiovascular diseases excluded from enrollment, and a lower cardiovascular surveillance in initial studies.^7^^,^^14^

According to ClinicalTrials.gov, in May 2024 there were 565 active trials starting or ongoing, which could exponentially increase the indications for CAR-T cell therapy for both hematological and solid tumors, autoimmune diseases and even in treatments of cardiomyopathies.^30^ Knowing the real prevalence of CVAE, increasing knowledge of which patients are prone to these adverse effects and the best way to address them becomes fundamental and urgent.

### Pre-therapy evaluation, monitoring and management

If the pivotal studies were unable to shed light on cardiotoxicity related to CAR-T cell therapy, subsequent cohort studies, and meta-analyses reinforced the need for integration between cardiology and oncohematology in the pre-therapy assessment and short- and long-term post-therapy monitoring. Infusion and early management of CRS and CVAE are needed to achieve full success in the therapy.

Part of a pre-therapy assessment is based on careful pre-treatment screening of cardiovascular comorbidities using a baseline electrocardiogram, chemistry panel including measurements of cardiac biomarkers (ultrasensitive troponin and natriuretic peptides), and transthoracic echocardiogram preferably with global longitudinal strain.^16^^,^^31^, ^32^, ^33^ Special attention should be paid to recipients who have conditions associated with increased risk for CVAE as described in Table 3, where, at the discretion of the multidisciplinary team's clinical judgment, additional complementary exams may be necessary and therapeutic optimization of cardiovascular morbidities carried out.^7^^,^^34^^.^ In a retrospective study, echocardiographic data such as higher mitral E/e’ and lower global longitudinal strain obtained from baseline echocardiogram in the pre-therapy evaluation were associated with a greater propensity for CVAE, thus allowing better surveillance in post-infusion follow-up. ^35^

**Figure 3:**
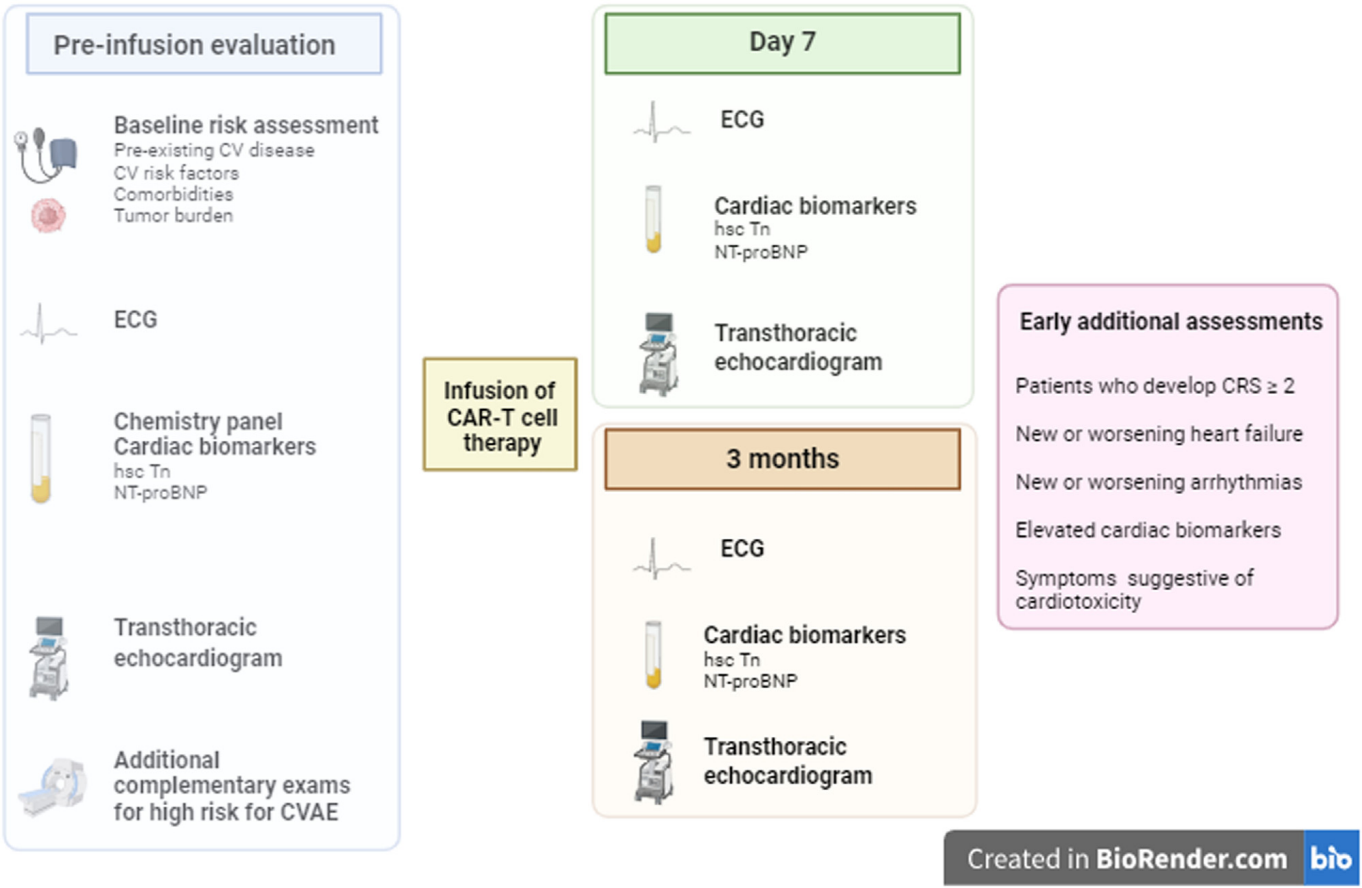
Pre-therapy evaluation and post infusion monitoring for cardiovascular adverse effects. CV: cardiovascular; ECG: electrocardiogram; hsc-Tn: high-sensitivity troponin; NT-proBNP: N-terminal pro-brain natriuretic peptide; CVAE: cardiovascular adverse effects; CRS: cytokine release syndrome.

After therapy infusion, cardiac biomarkers, electrocardiogram, and echocardiogram are part of cardiac surveillance. According to expert opinion, since to date there are no established guidelines regarding risk stratification of CAR T-cell recipients for early detection of CVAE, these tests should be repeated seven days and three months after the infusion. Additional assessments should be carried out early in patients who develop CRS ≥ 2, a sub-group at higher risk for CVAE, and in patients who develop new or worsening heart failure or new or worsening arrhythmias (Figure 3).^7^^,^^22^^,^^33^

Laboratory tests are investigated for markers related to cardiotoxicity and their early detection. On comparing to a group that did not present SCE, higher peak levels of IL-6, C-reactive protein, ferritin, and troponin were found to be possible markers of severe cardiovascular events (SCE) and non-relapse mortality.^36^ However, in an observational study, brain natriuretic peptide (BNP) levels, but not troponin I, were higher in patients with SCE.^37^

The approach to patients presenting with CVAE involves a multidisciplinary team. Depending on the severity, the necessary measures include intravenous fluids, vasopressor support, inotropic therapy, mechanical support, and arrhythmia control.

Tocilizumab, an anti-IL-6-receptor antagonist, is indicated in cases of moderate to severe CRS (CRS ≥ 2) or in patients with suspected acute cardiovascular complications. In cases refractory to IL-6 antagonists, steroids are recommended.^7^^,^^16^^,^^22^^,^^31^ According to a cohort registry, there was an important correlation between time from onset of CRS to administration of tocilizumab for the incidence of cardiovascular events, a composite of arrhythmias, decompensated heart failure, and cardiovascular death (1.7-fold increased risk with each 12-h period from onset of CRS).^24^

A more contemporary approach may reduce the incidence and severity of CRS and CVAE in patients who are candidates for CAR-T cell therapy. In a recently published prospective observational study with a total of 44 adult oncohematological patients evaluated, the incidence of CRS after CAR-T cell therapy was 52 % (24 episodes in 23 patients: 13 grade 1, 10 grade 2, and 1 grade 3, no grade 4). Only two patients with CVAE were reported, one case of heart failure with preserved ejection fraction and the other of atrial fibrillation.^38^ Although the lower incidence and severity of CRS and CVAE in this study are not completely understood, some hypotheses may explain this better presentation. One of these theories is based on the fact that CAR-T cell therapy was historically the third, fourth, or fifth line therapy during cancer treatment, however, with its excellent results, it has been indicated for less sick candidates. Another hypothesis is related to the earlier identification of CRS and CVAE with the indication of tocilizumab in earlier stages of these complications (mean time from CRS to tocilizumab administration of one day).

## Conclusions

It is rare in the history of medicine to have disruptive therapies being developed with such a positive impact on the prognosis of serious diseases similar to immunotherapy for oncology patients with CAR T-cell therapy for oncohematology patients being the best example. This benefit highlights perspectives for evaluating the therapy for other oncological diseases, such as solid or non-oncological tumors, for autoimmune diseases, and for the prevention of myocardial fibrosis.

The rapid expansion of indications needs to be accompanied by a better understanding of the adverse effects, including cardiotoxicity. However, prospective studies are lacking to define better preventive, monitoring, and treatment approaches to this complication, with the literature being based primarily on expert opinions. This gap must be closed to create better preventive and therapeutic approaches through prospective studies with large cohorts of patients and multidisciplinary integration where oncologists, oncohematologists, cardiologists, and other specialists should work together to expand the appropriate use of this new therapy.

## Conflicts of interest

The authors declare that they have no known competing financial interests or personal relationships that could have appeared to influence the work reported in this paper.
